# Antioxidant Activity of Extract and Its Major Constituents from Okra Seed on Rat Hepatocytes Injured by Carbon Tetrachloride

**DOI:** 10.1155/2014/341291

**Published:** 2014-02-27

**Authors:** Lianmei Hu, Wenlan Yu, Ying Li, Nagendra Prasad, Zhaoxin Tang

**Affiliations:** ^1^College of Veterinary Medicine, South China Agricultural University, Guangzhou 510642, China; ^2^Chemical Engineering Discipline, School of Engineering, Monash University, 46150 Bandar Sunway, Malaysia

## Abstract

The antioxidant activities and protective effects of total phenolic extracts (TPE) and their major components from okra seeds on oxidative stress induced by carbon tetrachloride (CCl_4_) in rat hepatocyte cell line were investigated. The major phenolic compounds were identified as quercetin 3-O-glucosyl (1 → 6) glucoside (QDG) and quercetin 3-O-glucoside (QG). TPE, QG, and QDG from okra seeds exhibited excellent reducing power and free radical scavenging capabilities including **α**, **α**-diphenyl-**β**-picrylhydrazyl (DPPH), superoxide anions, and hydroxyl radical. Overall, DPPH radical scavenging activity and reducing power of QG and QDG were higher than those of TPE while superoxide and hydroxyl radical scavenging activities of QG and TPE were higher than those of QDG. Furthermore, TPE, QG, and QDG pretreatments significantly alleviated the cytotoxicity of CCl_4_ on rat hepatocytes, with attenuated lipid peroxidation, increased SOD and CAT activities, and decreased GPT and GOT activities. The protective effects of TPE and QG on rat hepatocytes were stronger than those of QDG. However, the cytotoxicity of CCl_4_ on rat hepatocytes was not affected by TPE, QG, and QDG posttreatments. It was suggested that the protective effects of TPE, QG, and QDG on rat hepatocyte against oxidative stress were related to the direct antioxidant capabilities and the induced antioxidant enzymes activities.

## 1. Introduction

Oxidation is essential for living organisms. Reactive oxygen species (ROS) also are produced during oxidation [1]. Organisms can maintain a dynamic equilibrium between production and elimination of ROS in normal conditions. However, when organisms are subjected to stress conditions, this equilibrium is disrupted. Excessive accumulation of ROS will result in cellular injuries, including lipid peroxidation, protein oxidation, and DNA damage, which are involved in development of a variety of diseases including cellular aging, mutagenesis, carcinogenesis, hepatopathies, diabetes, and neurodegeneration [2]. Therefore, cellular antioxidant defense systems play important roles in counteracting these deleterious effects of ROS.

Almost all organisms possess antioxidant defense systems including antioxidant enzymes and nonenzymatic antioxidants. However, these systems are insufficient to prevent the damage entirely in some cases [3]. Plants are the most important source of natural antioxidants [4]. Phenolic compounds or polyphenols, which consist of secondary metabolites, constitute a wide and complex array of phytochemicals that exhibit antioxidant actions. Epidemiological studies have indicated that regular consumption of foods rich in phenolic compounds is associated with reduced risk of cardiovascular diseases, neurodegenerative diseases, and certain cancers [5, 6]. These phenolic compounds hold promising potentials in the development of health foods, nutritional supplements, and herbal medicines for the application as antioxidants and ROS-related disease chemopreventive agents.

Okra (*Hibiscus esculentus* L.), also known as lady's finger and gumbo, belongs to Malvaceae family, which is distributed widely in Africa, Asia, Southern Europe, and America [7]. The plant is a common vegetable in most regions for its nutrition value. Okra pod contains thick slimy polysaccharides, which are used to thicken soups and stews, as an egg white substitute, and as a fat substitute in chocolate bar cookies and in chocolate frozen dairy dessert [7]. Okra seed is rich in protein and unsaturated fatty acids such as linoleic acid [8]. In some countries, okra also is used in folk medicine as antiulcerogenic, gastroprotective, diuretic agents [9]. In addition, Arapitsas [10] reported that okra seed was rich in phenolic compounds, mainly composed of flavonol derivatives and oligomeric catechins, suggesting that it might possess some antioxidant properties. However, little information on antioxidant capabilities of major phenolic compounds from okra seed is available.

Carbon tetrachloride (CCl_4_), a well-known environmental biohazard, can be particularly toxic to liver. CCl_4_-induced hepatic injury, a classic experimental model, has been extensively used to evaluate the potential of drugs and dietary antioxidants against the oxidative damage [11, 12]. The objectives of the study were to evaluate the antioxidant activity of major phenolic compounds *in vitro* and their effects on oxidative stress induced by carbon tetrachloride (CCl_4_) in rat hepatocyte cell line.

## 2. Materials and Methods

### 2.1. Plant Materials

Okra pods (*Hibiscus esculentus* L.) were harvested from a commercial orchard in Guangzhou, Guangdong, China. The fruit were manually separated, and the seeds were collected, sun-dried, and pulverized to a powder. The materials were stored at room temperature in a desiccator until use.

### 2.2. Extraction, Isolation, and Purification

**Figure 1:**
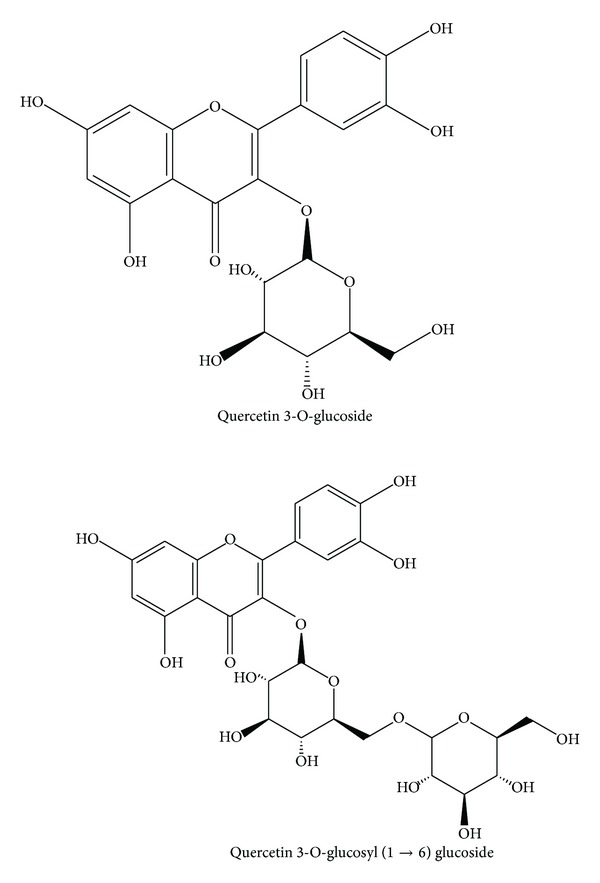
The chemical structures of the isolated compounds from *Hibiscus esculentus*. 1, quercetin 3-O-glucoside; 2, quercetin 3-O-glucosyl (1 → 6) glucoside.

Dried seed powder of *H. esculentus* was exhaustively extracted with methanol at temperature (25–32°C) for 3 days. The extracts were concentrated with a rotary evaporator (RE52AA, Yarong Equipment Co., Shanghai, China) under reduced pressure at 55°C and then fractionated sequentially by petroleum ether and EtOAc. The extraction with petroleum ether was to eliminate the pigments. EtOAc extract was obtained by evaporation under reduced pressure and then subjected to purification by silica gel column using CHCl_3_-MeOH solvent system with increased polarity (0 : 100–60 : 40) to yield eight fractions. We were only interested in the major phenolic compounds. Therefore, the largest fractions were further purified by silica gel column and Sephadex LH-20 to yield compound 1 and compound 2, respectively. Compound 1 and compound 2 were identified as quercetin 3-O-glucosyl (1 → 6) glucoside and quercetin 3-O-glucoside (Figure 1), by comparison of experimental and literature NMR data [13].

### 2.3. Determination of Total Phenol Content

Total phenol content of TPE in *H. esculentus* was determined by the Folin-Ciocalteu method [14]. Chlorogenic acid was used as a standard. The total phenol content was determined in triplicate and expressed as chlorogenic acid equivalents in mg/g of plant material.

### 2.4. Evaluation of Antioxidant Activities

Antioxidant capabilities of total phenolic extracts and their major component from okra were evaluated according to the described methods by Duan et al. [15] with minor modifications. To evaluate DPPH scavenging, 0.1 mL various concentrations of samples were mixed with 2.9 mL 0.1 mM DPPH-methanol solution. After 30 min of incubation at 25°C in the dark, the absorbance at 517 nm was measured. DPPH radical scavenging activity of the samples was calculated using the following formula: DPPH scavenging activity (%) = [1 − (absorbance  of  sample − absorbance  of  blank)/absorbance  of  control] × 100.

Superoxide radicals were generated by illuminating a solution containing riboflavin. The photoinduced reactions were performed at about 4000 lux. 25 *μ*L various concentrations of sample were mixed with 3 mL reaction buffer [1.3 *μ*M riboflavin, 13 mM methionine, 63 *μ*M nitroblue tetrazolium (NBT), and 100 *μ*M EDTA, pH 7.8]. The reaction solution was illuminated at 25°C for 15 min. The reaction mixture without sample was used as a control. The scavenging activity was calculated as follows: scavenging activity (%) = (1 − absorbance  of  sample/absorbance  of  control) × 100.

Hydroxyl radical scavenging activity was determined by evaluating inhibitory effect of samples on deoxyribose degradation. 0.2 mL different concentrations of samples were incubated with 1 mL reaction buffer (100 *μ*M FeCl_3_, 104 *μ*M EDTA, 1.5 mM H_2_O_2_, 2.5 mM deoxyribose, and 100 *μ*M L-ascorbic acid, pH 7.4) for 1 h at 37°C. After adding 1 mL 0.5% 2-thiobarbituric acid and 1 mL 2.8% trichloroacetic acid, the mixture was heated for 30 min at 80°C and then cooled on ice. The absorbance at 532 nm was measured. Percent inhibition of deoxyribose degradation was calculated as (1 − absorbance  of  sample/absorbance  of  control) × 100.

For the reducing power, 0.2 mL various concentrations of samples were incubated with 2.0 mL 200 mM sodium phosphate buffer (pH 6.6) and 2.0 mL 1% potassium ferricyanide at 50°C for 20 min. After adding 2.0 mL 10% trichloroacetic acid (w/v), the mixture was centrifuged at 4000 ×g for 5 min. 2.0 mL supernatant was mixed with 2.4 mL 0.016% ferric chloride, and the absorbance at 700 nm was measured. A higher absorbance indicates a higher reducing power.

### 2.5. Cell Culture and Cell Treatment

BRL-3A (rat hepatocyte) cell culture was obtained from Experimental Animal Center of Southern Medical University (Guangzhou, China). The cell line was cultured in RPMI-1640 medium containing 10% foetal bovine serum (FBS), 100 U/mL penicillin, and 100 mg/mL streptomycin at 37°C in an incubator of humidified air with 5% CO_2_. Hepatocytes were seeded onto 24-well plates at 1 × 10^5^ cells/well and cultured for 24 h at 37°C under 5% CO_2_. Later, the medium was removed and the hepatocytes were subjected to the following treatments: (1) pretreatment, the hepatocytes were first incubated with growth medium containing 100 *μ*g/mL TPE, QG, or QDG for 4 h, and then the cells were washed and incubated with fresh growth medium containing 10 mM CCl_4_ for another 4 h; (2) posttreatment, the hepatocytes were first incubated with growth medium containing 10 mM CCl_4_ for 4 h, and then the cells were washed and incubated with fresh growth medium containing 100 *μ*g/mL TPE, QG, or QDG for another 4 h.

### 2.6. Cell Viability

Cell viability of the hepatocytes was evaluated by MTT assay. After the cells were treated according to the above-mentioned method, the medium was removed and 20 *μ*L MTT solution (5 g/L) was added to each well of the 96-well plate. After 4 h of incubation at 37°C, the MTT solution was removed and 100 *μ*L dimethylsulphoxide (DMSO) was added to resolve the formazan generated from MTT. The absorbance of each well was recorded on a microplate reader (Thermo, MA, USA) at the wavelength of 490 nm. Cell viability in each test group and model group was expressed as percentage of the control group. The viability of cells in control group was considered as 100%.

### 2.7. MDA Content and Activities of GPT, GOT, SOD, and CAT

After the cells were treated according to the above-mentioned method, the supernatants were collected and assayed for malondialdehyde (MDA) content and activities of glutamate pyruvate transaminase (GPT), glutamate oxalate transaminase (GOT), superoxide dismutase (SOD), and catalase (CAT) using commercial enzymatic kits from Nanjing Jiancheng Bioengineering Research Institute (Nanjing, China).

### 2.8. Statistical Analysis

All data were expressed as mean ± standard deviation (SD). SPSS was used to analyze and report the data. The differences between the mean values of multiple groups were analyzed by one-way analysis of ANOVA with Duncan's Multiple Range Test. ANOVA data with *P* < 0.05 were classified as statistically significant.

## 3. Results and Discussions

### 3.1. Extraction of TPE and Purification of Major Constituent from *Hibiscus esculentus* Seed

*Hibiscus esculentus* seed was subjected to extraction with methanol and then sequential fractionation by petroleum ether and EtOAc. The extraction with petroleum ether was to eliminate the pigments. The EtOAc-soluble fraction was designated as total phenol extracts (TPE). The content of phenolic compounds in dry okra seed was 28.1 mg/g. Further, total phenol extracts (TPE) were purified and two major phenolic compounds were obtained and identified as quercetin 3-O-glucosyl (1 → 6) glucoside (QDG) and quercetin 3-O-glucoside (QG) (Figure 1), by comparison of experimental and literature NMR data [13], which was consistent with the result reported by Arapitsas [10]. However, Atawodi et al. reported that quercetin glucoside was the only major polyphenol composition in okra seed [16]. The inconsistence might be associated with the differences in climate conditions of cultivation and/or the variety analyzed.

### 3.2. Antioxidant Activity of Total Phenolic Extracts (TPE) and Their Major Components from Okra Seeds *In Vitro*

Free radical scavenging activity is the most important mechanism by which antioxidants inhibit lipid peroxidation [1]. In this study, free radical scavenging activity against DPPH radical, superoxide anions, and hydroxyl radical was analyzed to evaluate the antioxidant activities of total phenolic extracts (TPE) and their major components from okra seeds. In addition, reducing power, which exerts antioxidant action by breaking the free radical chain by donating a hydrogen atom, was investigated [15].

**Figure 2:**
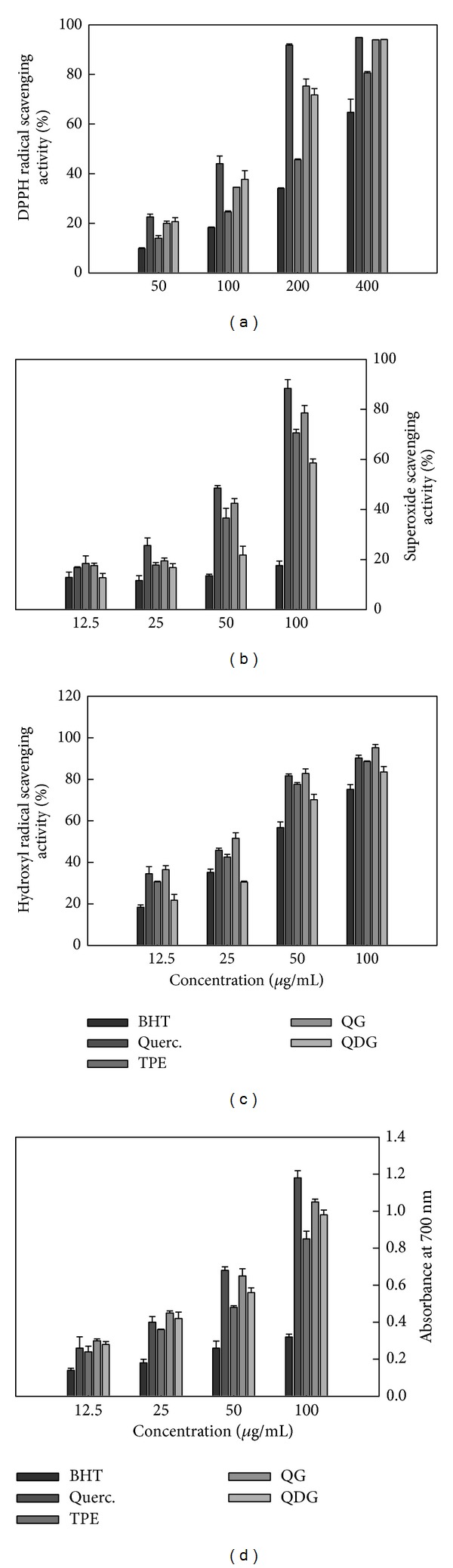
Free radical scavenging activities against DPPH radical (a), superoxide radical (b), and hydroxyl radical (c) and reducing power (d) of extract and isolated quercetin 3-O-glucoside and quercetin 3-O-diglucoside from *Hibiscus esculentus*.

DPPH is a stable, purple, nitrogen-centered, and synthetic-free radical with the maximum wavelength of 517 nm. When the DPPH free radical is quenched, the color will fade away. The scavenging activity against DPPH free radical has been extensively used to evaluate antioxidant activity of plant extracts [17].  Figure 2(a) shows the scavenging activities against DPPH free radical of TPE, QG, and QDG from okra seed and BHT and quercetin. All tested samples exhibited the scavenging activities against DPPH free radical in a dose-dependent manner. The positive control quercetin and BHT had the highest and lowest scavenging activity against DPPH free radical, respectively. QG and QDG have almost an equivalent scavenging activity against DPPH free radical, but higher than TPE. At 100 *μ*g/mL, the scavenging effects were 18.3%, 44.1%, 24.6%, 34.5%, and 37.3% for BHT, quercetin, TPE, QG, and QDG, respectively.

Superoxide anion, produced by a number of cellular reactions or enzymes, including iron-catalyzed Fenton reaction, lipoxygenases, peroxidase, NADPH oxidase, and xanthine oxidase, is the most important in organisms and is involved in the formation of other cell-damaging free radicals [1]. In the present study, TPE, QG, and QDG showed higher scavenging activity against superoxide anion than DPPH radical scavenging activity. Similarly, superoxide anion scavenging activities of TPE, QG, and QDG were stronger than that of BHT, but weaker than that of quercetin. At 50 *μ*g/mL and 100 *μ*g/mL of concentrations, the free radical scavenging activity of TPE was higher than that of QDG, but lower than that of QG (Figure 2(b))

Hydroxyl radical can be formed by the Fenton reaction in the presence of reduced transition metals and H_2_O_2_, which is known to be the most reactive radical and is thought to initiate cell damage *in vivo* [18]. Similar to superoxide anion scavenging activity, TPE, QG, and QDG from okra seeds exhibited excellent hydroxyl radical scavenging activity in the following order: QG > TPE > QDG > BHT (Figure 2(c)).

Reducing power is widely used to evaluate the antioxidant activity of plant extracts. The reducing property indicates that the antioxidant compounds are electron donors and reduce the oxidized intermediates of lipid peroxidation process [19]. As shown in Figure 2(d), TPE, QG, and QDG from okra seeds showed much higher reducing power than BHT, suggesting that TPE, QG, and QDG possessed a stronger electron donating capacity. However, the reducing power of TPE, QG, and QDG was lower than that of quercetin at 100 *μ*g/mL of concentration. Furthermore, the reducing power of the extract and its major constituents from okra seed were in the following order: QG > QDG > TPE.

Overall, DPPH radical scavenging activity and reducing power of QG and QDG were higher than those of TPE; superoxide and hydroxyl radical scavenging activities of QG and TPE were higher than those of QDG; reducing power, superoxide anion, and hydroxyl radical scavenging activities of QG were higher than those of QDG. Moreover, the antioxidant activities investigated, except hydroxyl radical scavenging activity of quercetin, were higher than those of QG and QDG. It appeared that addition of glycosyl decreased the scavenging activity against DPPH free radical of quercetin and its derivatives. Some similar results were obtained by other researchers. Omololu et al. reported that quercetin had stronger scavenging activity against DPPH free radical than its rhamnosyl glucoside derivative [20]. Hopia and Heinonen investigated the antioxidant activities of quercetin and its selected glycosides in bulk methyl linoleate oxidized at 40°C and found that the order of activity of quercetin and its derivatives was quercetin > isoquercitrin > rutin [21]. Sun et al. also found that rhamnosidase could change rutin in asparagus juice to quercetin-3-glucoside, which has a higher antioxidant activity than rutin [22]. The possible reasons are as follows: (1) the steric effect by increased glycosylation decreased the accessibility of free radicals to flavonoid antioxidants; (2) the presence of a free 3-hydroxyl group in the C-ring is a requirement for the maximal radical scavenging activity of flavonoids and the substitution of the hydroxyl group by glycoside resulted in the decreases in free radical scavenging ability and chelating activity to transition metal ions [21, 23]. In addition, QG and QDG played a dominant role in scavenging activity against superoxide anion and hydroxyl radical of total phenolic compounds of okra seed. In some plant flavonoids, quercetin 3-O-glucoside contributed to the major of antioxidant activities [24–27].

### 3.3. Effect of TPE, QG, and QDG on Cell Viability of Hepatocytes Injured by CCl_4_

**Figure 3:**
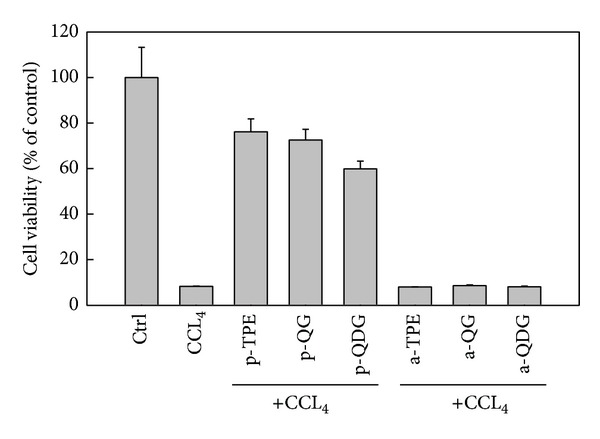
Effect of extract and isolated quercetin 3-O-glucoside and quercetin 3-O-diglucoside from *Hibiscus esculentus* on CCl_4_-induced cytotoxicity in rat hepatocytes. In the tick label, p means pretreatment while a means posttreatment.

It is well known that cell damage induced by reactive oxygen species (ROS) is an important mechanism of hepatotoxicity [28]. Tetrachloride (CCl_4_) has been widely used to study liver injury induced by ROS in the mouse model. The mechanism is involved in free radicals generated during CCl_4_ metabolism by hepatic cellular cytochrome P450, including trichloromethyl (CCl_3_ and/or CCl_3_O_2_) and oxygen-centered lipid radicals (LO and/or LOO), which initiate the process of lipid peroxidation [29]. Kikkawa et al. suggested that *in vitro *primary cell culture system would be sufficient to detect hepatotoxicity in the early stage of drug discovery according to the relevance of *in vitro* system to *in vivo* system from some biomarkers related to oxidative stress by carbon tetrachloride [12]. In the present study, CCl_4_ treatment resulted in a significant decrease in cell viability of the hepatocytes. After 4 h of incubation, the cell viability decreased to 8.3% compared with the control, indicating that the hepatocytes were severely injured. TPE, QG, and QDG pretreatments efficiently alleviated oxidative injury of the cell induced by CCl_4_. The cell viabilities of hepatocytes pretreated with TPE, QG, and QDG were 76.2%, 72.5%, and 59.9%, respectively (Figure 3). However, posttreatment with TPE, QG, and QDG had no significant protective effect on oxidative injury of the cells caused by CCl_4_. These results suggest that the extract and its major constituents can be potentially used for preventing rather than curing liver diseases in mammals. A similar result also was reported in fish hepatocytes by Yin et al. [30].

Antioxidants play an important role in protecting against CCl_4_-induced liver injury. There were some reports on the protective effects of various natural products against CCl_4_-induced liver injury [30–36]. The protective effects might be related to the directed superoxide anion and hydroxyl radical scavenging activity. In our study, superoxide anion and hydroxyl racial scavenging activities were in the following order: QG > TPE > QDG, which was inconsistent with the protective effects. Possibly, the discrepancy could be related to differential uptake by hepatocytes or synergistic effects. Boyer et al. [37] reported that there existed a great difference in uptake of quercetin aglycon and quercetin 3-glucoside as purified compounds and from whole onion and apple peel extracts by Caco-2 cells. Yang and Liu [38] also found that apple extracts plus quercetin 3-beta-d-glucoside combination possess a synergistic effect in MCF-7 cell proliferation.

### 3.4. Effect of TPE, QG, and QDG on Lipid Peroxidation and Activities of GPT and GOT in Hepatocytes Injured by CCl_4_

**Figure 4:**
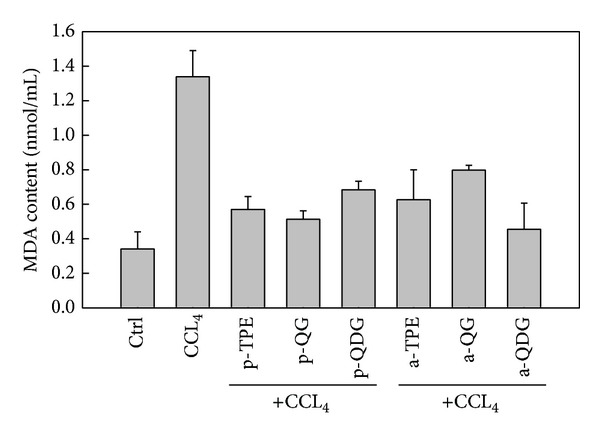
Effect of extract and isolated quercetin 3-O-glucoside and quercetin 3-O-diglucoside from *Hibiscus esculentus* on lipid peroxidation in the cell culture of hepatocytes injured by addition of CCl_4_ *in vitro*. In the tick label, p means pretreatment while a means posttreatment.

Malondialdehyde (MDA), a lipid peroxidized product, can reflect the extent of lipid peroxidation induced by oxidative stress. As shown in Figure 4, a significant increase in MDA level was observed in the CCl_4_-treated hepatocytes compared with the control hepatocytes. However, TPE, QG, and QDG treatments at 100 *μ*g/mL significantly decreased the level of lipid peroxidation induced by CCl_4_. The results were in accordance with the cell viability, indicating that the protective effects of TPE, QG, and QDG on CCl_4_-induced hepatocytes injury were related to the alleviated lipid peroxidation.

**Figure 5:**
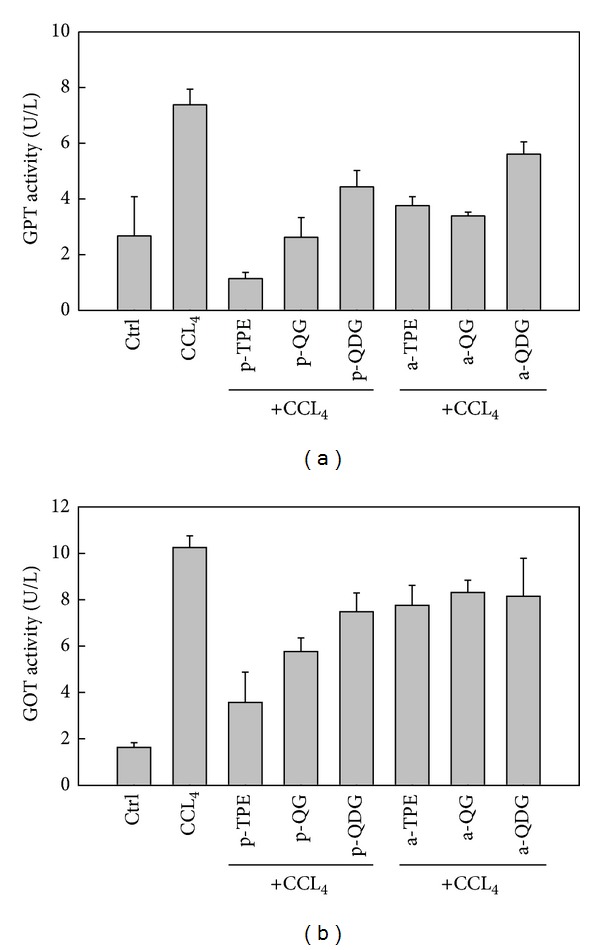
Effect of extract and isolated quercetin 3-O-glucoside and quercetin 3-O-diglucoside from *Hibiscus esculentus* on the activities of GPT (a) and GOT (b) in the cell culture of hepatocytes injured by addition of CCl_4_ *in vitro*. In the tick label, p means pretreatment while a means posttreatment.

GPT and GOT were widely used to evaluate liver damage by CCl_4_ [30]. In the present study, significantly elevated GPT and GOT activities were observed in the supernatants of the CCl_4_-treated hepatocytes, which might be associated with the increased permeability of the hepatocytes and cellular leakage. Pretreatments with TPE, QG, and QDG significantly decreased the values of GPT and GOT activities (Figure 5), indicating that the extract and its major constituents from okra seed could maintain the functional integrity of the hepatocyte membrane, thus protecting the hepatocytes against CCl_4_-mediated toxicity.

### 3.5. Effect of TPE, QG, and QDG on Activities of SOD and CAT in Hepatocytes Injured by CCl_4_

**Figure 6:**
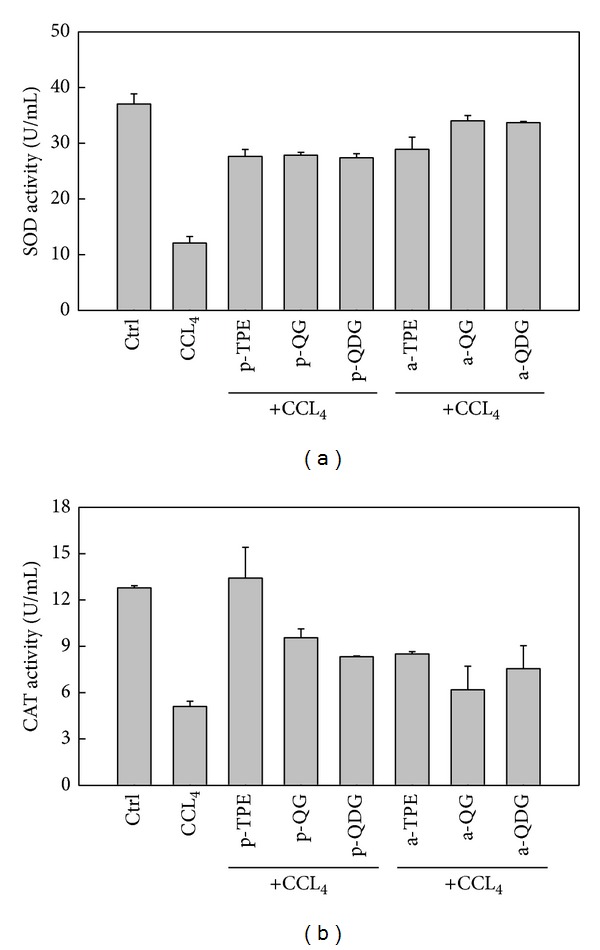
Effect of extract and isolated quercetin 3-O-glucoside and quercetin 3-O-diglucoside from *Hibiscus esculentus* on the activities of SOD (a) and CAT (b) in the cell culture of hepatocytes injured by addition of CCl_4_ *in vitro*. In the tick label, p means pretreatment while a means posttreatment.

Lipid peroxidation is the result of the excessive accumulation of ROS due to the altered balance between ROS generation and elimination in organism [39]. To control the level of ROS and protect cells against oxidative injury, organisms have developed an enzymatic antioxidant system and low molecular antioxidants [1]. Superoxide dismutase (SOD) is an important defense enzyme that catalyzes the dismutations of superoxide radicals to hydrogen peroxide while catalase (CAT) is involved in eliminating H_2_O_2_. As shown in Figure 6(a), CCl_4_ treatment alone resulted in a significant decrease in SOD activity of hepatocytes, as compared with the control. However, TPE, QG, and QDG pretreatments restored SOD activities in contrast to the CCl_4_-treated hepatocytes, which were beneficial in scavenging the superoxide anion and alleviating lipid peroxidation. There were no significant differences in SOD activities among TPE, QG, and QDG pretreated hepatocytes. Moreover, TPE, QG, and QDG posttreatments also induced SOD activities in the CCl_4_ pretreated hepatocytes (Figure 6(a)). Similarly, CAT activities in the CCl_4_-treated hepatocytes also were restored by TPE, QG, and QDG pretreatments. Even the same level of CAT activity, as compared with the control, was found in TPE-treated hepatocytes, which was higher than those in QG and QDG pretreated hepatocytes. CAT activities were partially restored by these treatments (Figure 6(b)).

Possibly, the alleviated biomacromolecule oxidation and elevated cell viability require synergic action of different antioxidant enzymes. There were some reports on the induced activities of antioxidant enzymes by natural products that played an important role in alleviating lipid peroxidation and decreasing the injury caused by CCl_4_ [30, 31, 34, 36]. In the present study, both SOD and CAT activities were induced by pretreatments or posttreatments with TPE, QG, and QDG. However, posttreatment with TPE, QG, and QDG had no significant protective effect on oxidative injury of the cells caused by CCl_4_. Therefore, it is considered that the induced SOD and CAT activities by posttreatment with TPE, QG, and QDG could not repair the damaged hepatocytes.

## 4. Conclusions

Two major flavonoids, quercetin 3-O-diglucoside (QDG) and quercetin 3-O-glucoside (QG), were isolated and identified from okra seeds. QG, QDG, and the total phenolic extracts (TPE) from okra seeds showed excellent antioxidant activity *in vitro*, including reducing power and free radical scavenging capabilities against *α*,*α*-diphenyl-*β*-picrylhydrazyl (DPPH) radical, superoxide anion, and hydroxyl radical, which were much stronger than those of BHT, a widely used synthetical antioxidant. DPPH radical scavenging activity and reducing power of QG and QDG were higher than those of TPE while superoxide and hydroxyl radical scavenging activities of QG and TPE were higher than those of QDG. Moreover, reducing power, superoxide anion, and hydroxyl radical scavenging activities of QG were higher than those of QDG. Furthermore, TPE, QG, and QDG pretreatments significantly alleviated the cytotoxicity of CCl_4_ on rat hepatocytes, with attenuated lipid peroxidation, increased SOD and CAT activities, and decreased GPT and GOT activities. The protective effects of TPE and QG on rat hepatocytes were stronger than that of QDG. However, the cytotoxicity of CCl_4_ on rat hepatocytes was not affected by TPE, QG, and QDG posttreatments. It was suggested that the protective effects of TPE, QG, and QDG on rat hepatocyte against oxidative stress were related to the direct antioxidant activity and the induced activities of antioxidant enzymes. However, further investigation is required to evaluate protective effects of total phenolic extracts (TPE) and their major components from okra seeds on carbon tetrachloride-induced hepatotoxicity *in vivo*.
